# Self-Assembled Vanadium Oxide Nanoflakes for p-Type Ammonia Sensors at Room Temperature

**DOI:** 10.3390/nano9030317

**Published:** 2019-02-27

**Authors:** Haihong Yin, Changqing Song, Zhiliang Wang, Haibao Shao, Yi Li, Honghai Deng, Qinglan Ma, Ke Yu

**Affiliations:** 1 School of Information Science and Technology, Nantong University, Nantong 226019, China; hhyin@ntu.edu.cn (H.Y.); cqsong@ntu.edu.cn (C.S.); haibao@ntu.edu.cn (H.S.); liyi2016@ntu.edu.cn (Y.L.); denghonghai@ntu.edu.cn (H.D.); maqinglan@ntu.edu.cn (Q.M.); 2 Key Laboratory of Polar Materials and Devices, Department of Optoelectronics, East China Normal University, Shanghai 200241, China; yk5188@263.net; 3 Collaborative Innovation Center of Extreme Optics, Shanxi University, Taiyuan 030006, China

**Keywords:** vanadium oxides, self-assembled nanoflakes, ammonia, gas sensors

## Abstract

VO_2_(B), VO_2_(M), and V_2_O_5_ are the most famous compounds in the vanadium oxide family. Here, their gas-sensing properties were investigated and compared. VO_2_(B) nanoflakes were first self-assembled via a hydrothermal method, and then VO_2_(M) and V_2_O_5_ nanoflakes were obtained after a heat-phase transformation in nitrogen and air, respectively. Their microstructures were evaluated using X-ray diffraction and scanning and transmission electron microscopies, respectively. Gas sensing measurements indicated that VO_2_(M) nanoflakes were gas-insensitive, while both VO_2_(B) and V_2_O_5_ nanoflakes were highly selective to ammonia at room temperature. As ammonia sensors, both VO_2_(B) and V_2_O_5_ nanoflakes showed abnormal p-type sensing characteristics, although vanadium oxides are generally considered as n-type semiconductors. Moreover, V_2_O_5_ nanoflakes exhibited superior ammonia sensing performance compared to VO_2_(B) nanoflakes, with one order of magnitude higher sensitivity, a shorter response time of 14–22 s, and a shorter recovery time of 14–20 s. These characteristics showed the excellent potential of V_2_O_5_ nanostructures as ammonia sensors.

## 1. Introduction

In many industries, hazardous gases have become increasingly important raw materials, and for this reason it has become very important to develop highly sensitive gas sensors to monitor them in the manufacturing process. To this end, finding suitable materials with the required surface/bulk properties is essential for the gas sensor development. So far, metal oxide semiconductors (MOS) are the most attractive gas-sensitive materials [1]. Because nanostructured materials have a large surface-to-volume ratio and abundant surface states, many gas sensors based on SnO_2_ [2,3,4], TiO_2_ [5,6], ZnO [2,7,8,9], WO_3_ [10,11], In_2_O_3_ [12,13,14], and other metal oxide nanostructures have been successfully fabricated to detect and quantify gaseous species. MOS sensors are applicable in many areas of human activity. Different MOS gas sensors can be used to construct sensor matrixes named electronic noses to identify different odorous compounds and quantify their concentration [15,16,17,18,19]. Among the numerous metal oxides, vanadium oxides form an interesting material group because of their varying oxidation states between V^2+^ and V^5+^ [20]. At least 15 different vanadium oxides (V_x_O_y_), such as VO, V_2_O_3_, VO_2_, V_2_O_5_, V_n_O_2n−1_, and V_2n_O_5n−2_, have been reported [21,22]. Among them, VO_2_ (B phase and M/R phase) and V_2_O_5_ are the most famous, attracting great interest due to their special chemical/physical properties and their potential application in many fields. Table 1 shows the crystal structure, properties, and some important applications of the three vanadium oxides. B phase VO_2_ and V_2_O_5_ both have a layered structure. The spacing between layers provides abundant sites for the facile intercalation of Li^+^, thus making them an attractive cathode material in lithium-ion batteries [23,24]. M-phase VO_2_ shows a semiconductor-to-insulator transition around 340 K, accompanied by a rapid change in resistivity and optical transmittance, thus exhibiting wide potential in ultrafast optical switches, thermochromic windows, and infrared sensors [25,26].

Recently, vanadium oxides have been considered as new candidates for gas sensors. Their sensing properties for inorganic/organic gases such as nitrogen oxides [27,28], ethanol [1,29,30,31], butyl-amine [32], and ammonia [33,34,35] have been reported. However, the gas-sensing properties of vanadium oxide nanostructures are strongly dependent on the actual synthesis environment and closely correlated with material morphologies, surface states, and microstructures. For example, several groups reported that V_2_O_5_ nanostructures (e.g. nanorods, flow-like nanostructures) were more sensitive to ethanol than to ammonia at room temperature [29,30,31]; however, an opposite higher sensitivity to ammonia is also reported by Hakim et al. in V_2_O_5_ nanoneedles [36]. Some groups even reported ultrahigh sensitivities of V_2_O_5_ nanostructures (e.g. nanofibers, nanofilms) for ultralow level (<1 ppm) ammonia detection [33,34,35]. In addition, the conduction type n or type p usually determines the direction of resistance change when they are exposed to target gases. When n-type MOS gas sensors are utilized to detect reducing gases, reductive gas species react with the chemisorbed oxygen on the surface and electrons trapped by oxygen are released into the conduction band of MOS, leading to a decrease in resistance. With regard to p-type MOS gas sensors, the direction of resistance change is opposite due to the combination of holes with electrons released from the surface reaction. Vanadium oxides’ nanostructures are generally regarded as n-type semiconductors, which exhibit n-type gas-sensing responses in most literature reports. However, interesting p-type sensing behaviors have also been reported in a few studies. Yu et al. reported a p-type sensing response to NO_2_ in vanadium oxide nanotubes at 80 °C [37]. Also, p-type sensing responses to either ethanol or ammonia at room temperature were reported in V_2_O_5_ and VO_2_(B) nanostructures [29,38]. Obviously, the synthesis environment and the microstructure played a crucial role in gas sensors of vanadium oxide nanostructures.

In this work, self-assembled VO_2_(B), VO_2_(M), and V_2_O_5_ nanoflakes were synthesized and their gas-sensing properties were comparatively investigated. VO_2_(B) nanoflakes were first self-assembled via a hydrothermal method, and then V_2_O_5_ and VO_2_(M) specimens were obtained by annealing VO_2_(B) nanoflakes in air and nitrogen, respectively. Their gas-sensing properties demonstrated that VO_2_(B) and V_2_O_5_ specimens displayed superior selectivity to ammonia, while the VO_2_(M) specimen was gas-insensitive. Unlike the common n-type gas-sensing mechanism most reported, both VO_2_(B) and V_2_O_5_ nanoflakes exhibited abnormal p-type sensing properties in ammonia. Compared to the VO_2_(B) specimen, the ammonia sensor of V_2_O_5_ nanoflakes showed a one order of magnitude higher sensitivity, faster response speed, and better reproducibility, exhibiting wide potential in ammonia sensors.

## 2. Materials and Methods

### 2.1. Synthesis of Self-Assembled Vanadium Oxide Nanoflakes

Self-assembled VO_2_(B) nanoflakes were prepared via a hydrothermal method, as described in previous works [39]. Briefly, 0.48 g commercial V_2_O_5_ powder was added to 80 mL oxalic acid (0.1 mol/L) in an aqueous solution to form a yellow slurry. The slurry was stirred for 30 min and then transferred to a 100-mL autoclave with a Teflon liner. The autoclave was maintained at 180 °C for 36 h and then air-cooled to room temperature. The resulting dark blue precipitates (VO_2_(B)) were collected and washed with distilled water and ethanol several times and then dried at 60 °C under a vacuum for 10 h. Finally, the VO_2_(M) specimen was obtained by heating VO_2_(B) at 550 °C in an N_2_ atmosphere for 1 h, and the V_2_O_5_ specimen was obtained by heating VO_2_(B) at 550 °C in air for 1 h. In all reactions, all chemicals used were purchased without further purification and without adding any additives or surfactants.

### 2.2. Characterization

The morphology of vanadium oxide specimens was characterized by field-emission scanning electron microscopy (FESEM, Hitachi-S-4800, Hitachi High Technologies, Tokyo, Japan) and transmission electron microscopy (JEOL, JEM-2100, Tokyo, Japan). The crystal structure was characterized by X-ray diffraction (XRD, Bruker D8 Advance, Billerica, MA, USA) using monochromatized Cu Kα radiation (λ = 1.5418Å).

### 2.3. Sensor Fabrication and Gas-Sensing Tests

**Figure 1:**
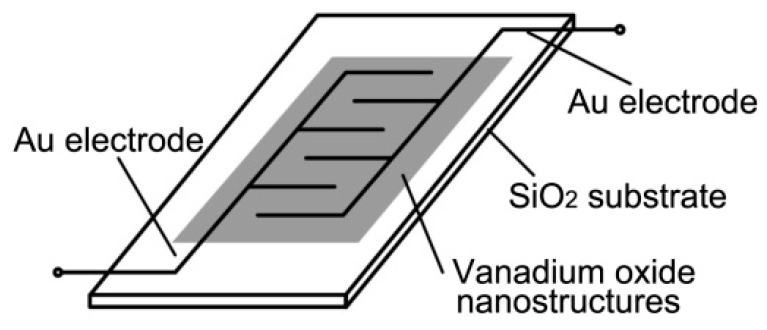
A schematic of the sensor substrate, the patterned gold electrodes and the sensing layer.

The diagram of gas sensors is shown in Figure 1. Synthesized vanadium oxide samples were deposited on a SiO_2_ tile for gas sensing tests. The SiO_2_ tile was pre-patterned with interdigitated gold electrodes. For each sensor fabrication, the individual substrate was placed in a hot plate and heated to 50 °C. VO_2_(B), VO_2_(M), and V_2_O_5_ samples were each separately dispersed in ethanol at a concentration of 200 mg mL^−1^ and then deposited on the hot sensor substrates. Finally, the sensor substrates with vanadium oxide samples across the interdigitated gold electrodes were dried in air for 40 min.

The gas-sensing properties of sensors were evaluated on a measuring system containing a static gas distribution chamber with a volume of 20 L. To simulate the actual measuring circumstances, we referenced the measuring method used by Qin et al. [29]. The ambient air was introduced into the chamber and target gases/liquids were injected and diluted/volatilized in the chamber. The relative chamber humidity was monitored using an internal humidity meter. During the measurement, the relative humidity is about 37% and the room temperature is about 24 °C.

## 3. Results

**Figure 2:**
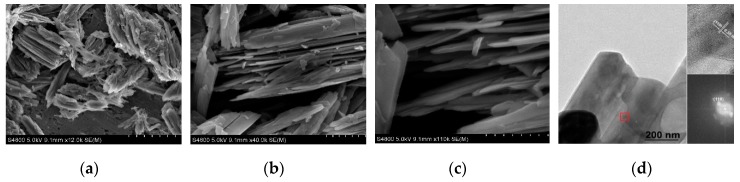
(**a**) Low-, (**b**) medium-, and (**c**) high-magnification SEM images of self-assembled VO_2_(B) nanoflakes synthesized by hydrothermal method. (**d**) TEM image of a single nanoflake. Upper inset: the high-resolution (HR) TEM image for the red square region. Lower inset: the corresponding fast Fourier transform (FFT) pattern.

VO_2_(B) specimen was prepared via a hydrothermal procedure, and their representative SEM images are shown in Figure 2. Obviously, the VO_2_(B) specimen consists of large quantities of nanoflakes. It is noted that these nanoflakes are not distributed randomly in the products, but parallel arranged and self-assembled forming clumps as shown in Figure 2a. The medium- and high-magnification SEM images indicate that these nanoflakes are typically several micrometers in length, hundreds of nanometers in width (Figure 2b), and 20–50 nm in thickness (Figure 2c). In addition, these nanoflakes are also assembled together in the TEM image (Figure 2d). The corresponding high-resolution (HR) TEM image (Figure 2d, square region marked in red) indicates that the nanoflakes are single crystalline, and the lattice spacing of 0.35 nm corresponds to the *d* spacing of the (110) plane of monoclinic VO_2_ (B). The corresponding fast Fourier transform (FFT) pattern (the inset of Figure 2d) indicates that these nanorods grew along the [110] direction.

**Figure 3:**
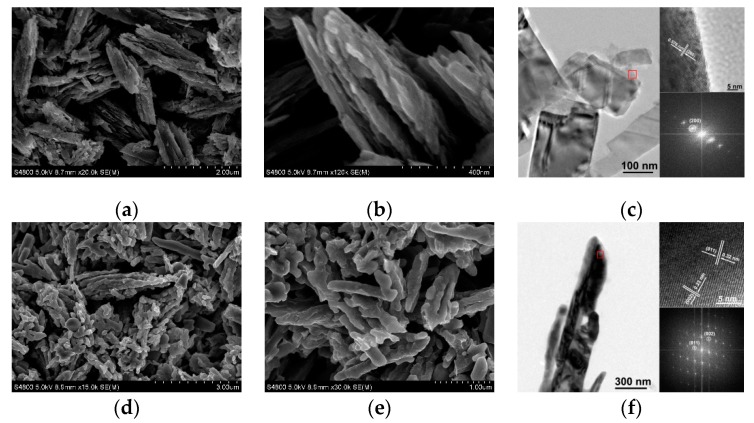
(**a**) Low- and, (**b**) high-magnification SEM images of V_2_O_5_ specimen obtained after annealing VO_2_(B) nanoflakes in air. (**c**) TEM images of V_2_O_5_ nanoflakes. (**d**) Low- and, (**e**) high-magnification SEM images of VO_2_(M) specimen obtained after annealing VO_2_(B) nanoflakes in nitrogen atmosphere. (**f**) TEM images of VO_2_(M) nanoflakes. Upper inset in plane (**c**) and (**f**): the high-resolution TEM image for the red square region. Lower inset in plane (**c**) and (**f**): the corresponding fast Fourier transform (FFT) pattern.

By oxidizing self-assembled VO_2_(B) nanoflakes in air at 550 °C, a V_2_O_5_ specimen was obtained. As shown in Figure 3a,b, the V_2_O_5_ specimen displays self-assembled flake-like nanostructures similar to the VO_2_(B) specimen. However, these V_2_O_5_ nanoflakes were slightly cracked after the calcination, showing a surface roughness increment in Figure 3b compared to Figure 2b, although they are also self-assembled (Figure 3a). The TEM image indicates that the self-assembled structure was destroyed after an ultrasonic treatment, forming randomly dispersed nanoflakes (Figure 3c). The HRTEM image (upper inset of Figure 3c) for a single nanoflake (region marked in a red square of Figure 3c) and the corresponding FFT patterns (lower inset of Figure 3c) show that the nanoflakes are single phase crystalline and the lattice spacing of 0.576 nm corresponds to the (200) plane of V_2_O_5_. In addition, by annealing VO_2_(B) nanoflakes in nitrogen at 550 °C, a VO_2_(M) specimen was obtained. As shown in Figure 3d,e, VO_2_(B) nanoflakes transform to rod- and particle-like VO_2_(M) nanostructures and the self-assembled flake-like structure is destroyed to some extent. The corresponding HRTEM image (upper inset of Figure 3f) indicates clear lattice fringes. The spacing between two adjacent lattice planes is 0.32 and 0.23 nm, respectively, corresponding to the (011) and the (002) plane of M phase VO_2_. The corresponding FFT patterns (lower inset of Figure 3f) confirm the single-crystalline nature of these VO_2_(M) nanostructures.

**Figure 4:**
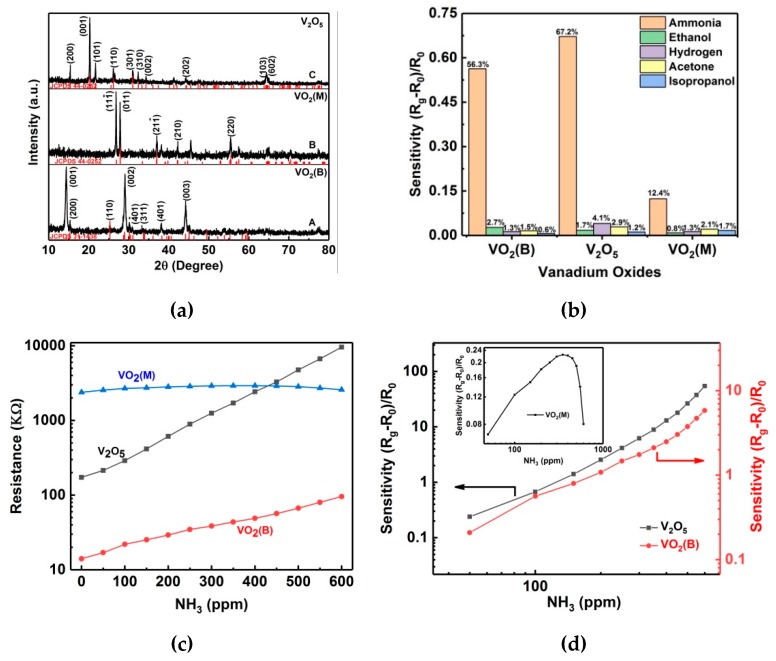
(**a**) XRD patterns of self-assembled VO_2_(B), VO_2_(M) and V_2_O_5_ specimens. (**b**) Selectivity of sensors based on VO_2_(B), V_2_O_5_ and VO_2_(M) nanoflakes for 100 ppm ammonia, ethanol, hydrogen, acetone and isopropanol at room temperature. (**c**) Sensor resistance variation with 50–600 ppm ammonia gas for VO_2_(B), VO_2_(M) and V_2_O_5_ self-assembled nanoflakes. (**d**) The sensitivity of sensors based on VO_2_(B) and V_2_O_5_ self-assembled nanoflakes at room temperature. *R_0_* and *R_g_* are the resistances in the air and the measured ammonia level respectively. The inset in plane (**d**) is the sensitivity for the sensor of VO_2_(M) specimen.

XRD patterns for the three specimens are shown in Figure 4a, where the patterns A, B and C correspond to the VO_2_(B), VO_2_(M), and V_2_O_5_ specimens, respectively. All peaks in pattern A are indexed to VO_2_(B) phase (space group: C2/m) with lattice constants of *a* = 12.03 Å, *b* = 3.693 Å, *c* = 6.42 Å, and *β* = 106.6^°^ (JCPDS 31–1438); no any other phases or impurities were detected, revealing that the V^5+^ ions in V_2_O_5_ have been reduced to V^4+^ ions by the oxalic acid in the hydrothermal reaction and the products are mainly composed of VO_2_(B). After the calcination in either nitrogen or air, the B phase VO_2_ transformed to VO_2_(M) and V_2_O_5_, respectively. As shown in Figure 4a, all peaks in patterns B and C are indexed to VO_2_(M) phase (space group P2_1_/c, JCPDS 44-0252) and orthorhombic V_2_O_5_ (space group Pmmn (59), JCPDS 41-1426), respectively, confirming the successful phase transform to VO_2_(M) and V_2_O_5_. No peaks of any other phases or impurities were detected in patterns B and C, revealing the high phase purity of VO_2_(M) and V_2_O_5_ products.

VO_2_(B), VO_2_(M), and V_2_O_5_ are the most famous compounds in the vanadium oxide family. In this work, their gas-sensing properties are investigated and compared. When sensors based on VO_2_(B), VO_2_(M), and V_2_O_5_ specimens are exposed in 100 ppm ammonia, ethanol, hydrogen, acetone, and isopropanol, they show different resistance response. As shown in Figure 4b, all vanadium oxide specimens exhibit higher response to ammonia, while no obvious response towards other target gases is observed. The resistance increment in 100 ppm ammonia are of 56.3%, 67.2%, and 12.4%, corresponding to VO_2_(B), V_2_O_5_, and VO_2_(M), respectively; thus, the three sensors show higher selectivity for ammonia than for other target gases. Figure 4c shows the resistance variation of VO_2_(B), VO_2_(M), and V_2_O_5_ sensors when they are exposed in 50–600 ppm ammonia at room temperature. Figure 4d and the inset show their corresponding sensitivity. It is noted that an approximated power-law dependence between the sensitivity and the NH_3_ concentration is observed for both V_2_O_5_ and VO_2_(B) nanoflakes, matching the sensor response law developed by Gurlo et al. [40]. Here, the sensitivity is defined as $S=\left(R_{g}-R_{0}\right)/R_{0}$, where $R_{0}$ and $R_{g}$ are the resistance of sensors in air and in ammonia, respectively. The resistance $R_{0}$ for sensors of VO_2_(B), VO_2_(M) and V_2_O_5_ are $\left(1.41\pm 0.063\right)\times 10^{4}$ Ω, $\left(2.38\pm 0.078\right)\times 10^{6}$ Ω, and $\left(1.73\pm 0.059\right)\times 10^{5}$ Ω, respectively. The difference in resistance can be interpreted by their different intrinsic electrical properties. V_2_O_5_ is a semiconducting/insulating oxide with a band gap of 2.2 eV, and usually exhibits low conductivity due to the empty 3d orbital [41]. VO_2_(M) undergoes a near room-temperature MIT accompanied by a rapid change in resistivity, usually showing high resistivity at room temperature [41]. Although VO_2_(B) is generally regarded as an n-type semiconductor in gas sensing research [38], it is a theoretical semimetal/metal phase at room temperature, usually exhibiting high conductivity compared to VO_2_(M) and V_2_O_5_ [42]. As the ammonia concentration increases from 0 to 600 ppm, both the VO_2_(B) and V_2_O_5_ sensors show nonlinear increases of resistance. The sensor resistance of VO_2_(B) in 600 ppm ammonia is $\left(9.58\pm 0.043\right)\times 10^{4}$ Ω, showing a ~5.80-fold increment, while the value for the V_2_O_5_ sensor is $\left(9.58\pm 0.067\right)\times 10^{6}$ Ω, showing a ~54.4-fold increment. Obviously, the sensor sensitivity of V_2_O_5_ is superior, about one order of magnitude higher than the sensitivity of VO_2_(B). Unlike sensors of VO_2_(B) and V_2_O_5_, the resistance of the VO_2_(M) sensor first increases with the ammonia concentration, reaches a maximum value of $\left(2.92\pm 0.033\right)\times 10^{6}$ Ω at 350 ppm, and then gradually decreases to $\left(2.5\pm 0.028\right)\times 10^{6}$ Ω at 600 ppm. Therefore, the VO_2_(M) sensor whose resistance and sensitivity fluctuate in a narrow range (Figure 4c and the inset of Figure 4d) is not suitable for the ammonia detection.

**Figure 5:**
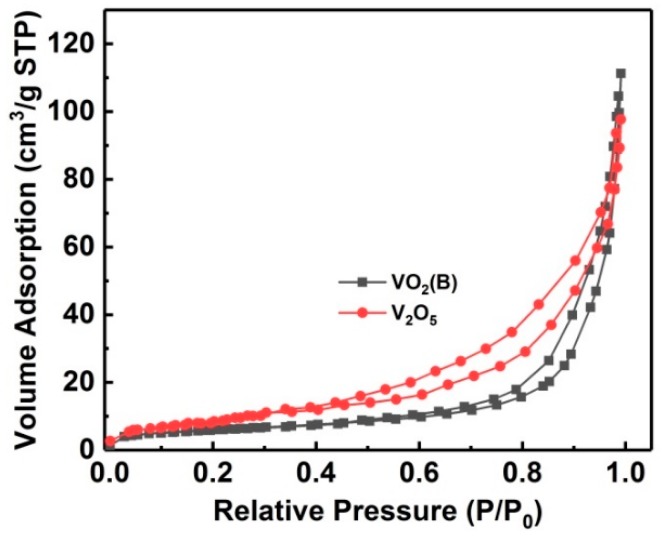
N_2_ adsorption–desorption isotherms of self-assembled VO_2_(B) and V_2_O_5_ nanoflakes.

To investigate the influence of the specific surface area on the ammonia sensing performance, we used the BET method of adsorption and desorption of nitrogen gas to measure the specific surface area of VO_2_(B) and V_2_O_5_ specimens. As shown in Figure 5, isotherms for both VO_2_(B) and V_2_O_5_ nanoflakes followed a typical IV-type curve with a clear hysteresis loop at *p*/*p*_0_ values of 0.55–0.99 for VO_2_(B) and 0.40–0.99 for V_2_O_5_. The BET specific surface area calculated from the nitrogen isotherms is 23.8 and 30.3 m^2^ g^−1^, corresponding to VO_2_(B) and V_2_O_5_ nanoflakes, respectively. Although V_2_O_5_ nanoflakes show a larger specific surface area, the two values are comparable, while the sensitivity of V_2_O_5_ in this work is about 10 times that of VO_2_(B). Moreover, V_2_O_5_ always showed higher sensing performance to ammonia than VO_2_(B) in the previous literature [38,43,44,45]. Therefore, one reasonable explanation is that the intrinsic properties of V_2_O_5_ determine its higher sensing performance. VO_2_(B) and V_2_O_5_ are generally regarded as n-type semiconductors in gas-sensing investigations [38]; however, VO_2_(B) is a theoretical semimetal/metal phase at room temperature [42]. Therefore, V_2_O_5_ should provide more absorbed oxygen ions on the surface, leading to high sensing performance.

The surface-depletion model is usually used to describe the sensing mechanism of the resistance-type metal-oxide semiconductor sensor. When the sensor is exposed to air, some oxygen molecules will be adsorbed on the surface, and then some oxygen ions, $\left(O^{-}{}_{\left(\mathrm{ads}\right)}\right)$, will be formed at the surface. When the sensor is put in the reducing gas, ammonia in this work, the reducing gas molecules will react with the oxygen ions on the surface as described below:

$$2{\mathrm{NH}}_{3}+3\left(O^{-}{}_{\left(\mathrm{ads}\right)}\right)\to N_{2}+3H_{2}O+3e^{-}.$$

Thus the carrier concentration changes with the ammonia level and, consequently, causes the sensor resistance to change. It is well known that semiconductor metal oxides are usually classified as either n-type or p-type. Conductivity type plays an important role in sensor responses. For n-type semiconductor sensors, the reductive gas species reacts with the adsorbed oxygen ions and the electrons trapped by oxygen are released into the conduction band, leading to a decrease in resistance. With regard to p-type semiconductor sensors, the opposite change in the resistance is observed due to the combination of holes with electrons released from the surface reaction. Both VO_2_(B) and V_2_O_5_ are generally considered to be n-type semiconductors in gas-sensing investigations, and there are many studies reporting their n-type gas-sensing responses. In studies by Raj et al. [31] and Modafferi et al. [34], sensors based on V_2_O_5_ displayed a resistance decrease when they were exposed to an ascending ammonia level and the material was treated as n-type. However, our measured results indicate that the sensor resistance of VO_2_(B) and V_2_O_5_ displays a unidirectional increase as the ammonia level increases from 0 to 600 ppm, showing typical p-type resistance responses. Similar intriguing p-type gas-sensing behaviors have been reported previously. Qin et al. [29] and Evans et al. [38] reported p-type resistance responses in V_2_O_5_ hierarchical networks and VO_2_(B) nanoparticles, and attributed them to the inversion layer formed at the surface due to a larger quantity of surface oxygen vacancy [29]. These contrasting results suggest that the sensing response type of vanadium oxides at room temperature is highly dependent on the synthesis environment and the resultant surface species.

**Figure 6:**
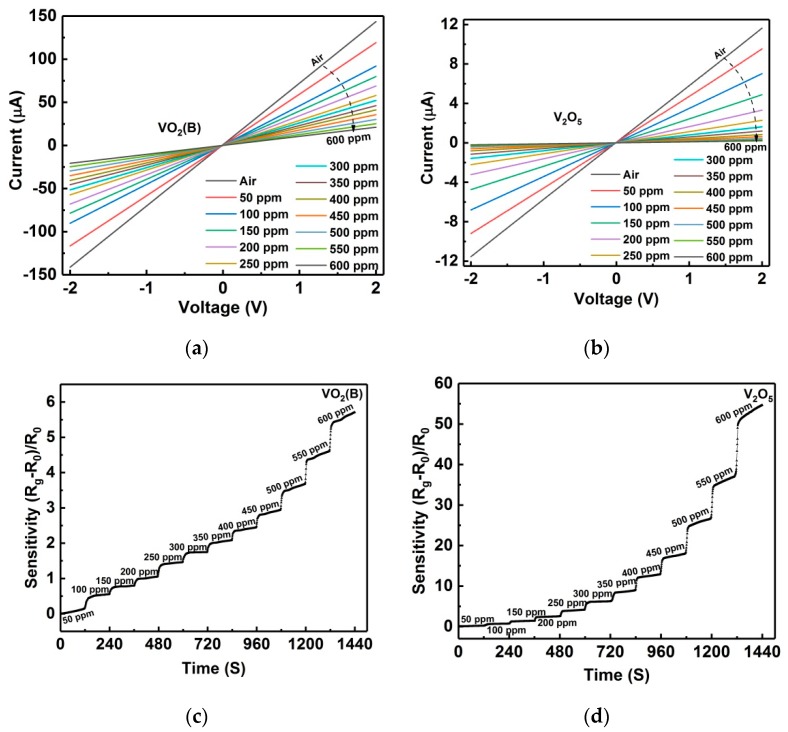
Room temperature I-V curves for sensors of (**a**) VO_2_(B), and (**b**) V_2_O_5_ nanoflakes measured in different static ammonia atmosphere from 0 to 600 ppm. Sensor responses of (**c**) VO_2_(B) and (**d**) V_2_O_5_ nanoflakes to ascending ammonia levels step-by-step.

I‒V curves for sensors of VO_2_(B) and V_2_O_5_ in 0–600 ppm ammonia are measured at room temperature and shown in Figure 6a,b. All of the curves appear to be linear, indicating that good ohmic contacts formed between the metal electrodes and nanoflakes. From the I‒V curves, it is seen that the conduction of two devices progressively decays with the ascending ammonia level, showing p-type sensing responses and agreeing with the resistance measurement (Figure 4c). Figure 6c,d present the dynamic responses of VO_2_(B) and V_2_O_5_ sensors in the ascending ammonia levels. When the ammonia was continuously injected into the testing chamber with a step of 50 ppm, both sensors showed nonlinear increases in their resistances. From Figure 6c,d, it is obvious that in each ammonia level of both sensors the resistance ladder is very clear, revealing good dynamic response characteristics. Moreover, the V_2_O_5_ sensor shows a one order of magnitude higher sensitivity than the VO_2_(B) sensor. In both sensors, NH_3_ reacts with the surface adsorbed oxygen ions, releasing electrons and inducing an electrical response to the ascending ammonia level. However, VO_2_ and V_2_O_5_ are normally considered n-type semiconductors at room temperature, so the formation of a surface inversion layer is a reliable interpretation for the p-type behaviors [29,38], where the released electrons would reduce the number of holes (the majority charge carriers in the inversion layer) and result in a p-type increase of the sensor resistivity.

**Figure 7:**
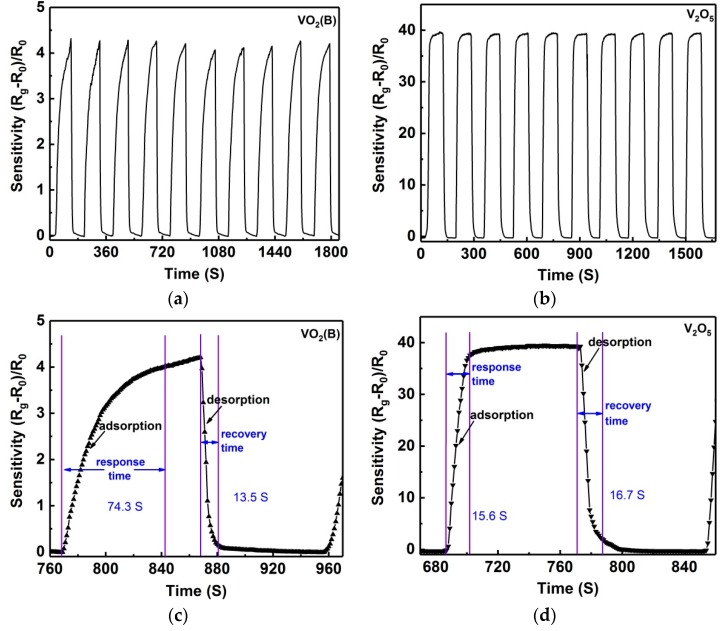
Dynamic responses for sensors of (**a**) VO_2_(B), and (**b**) V_2_O_5_ nanoflakes. Dynamic switches are performed between air and 550 ppm ammonia. (**c**,**d**) High-magnification dynamic responses for one cycle, corresponding to VO_2_(B)- and V_2_O_5_-based sensors.

Further dynamic testing procedures were carried out, which provided more information on the most important parameters for a sensing device: sensitivity, response and recovery time, and reproducibility. Figure 7a,b show the dynamic responses of VO_2_(B) and V_2_O_5_ sensors for 10 cycles where the dynamic mesurement was performed between air and 550 ppm ammonia at room temperature. For clear observation, only 10 cycles are given. Similarly, both VO_2_(B) and V_2_O_5_ sensors indicate good reproducibility, as revealed in the repeated measurements (Figure 7a,b). When sensors of VO_2_(B) and V_2_O_5_ were exposed to 550 ppm ammonia, the ammonia adsorption was triggered, and simultaneously the sensor resistance increased abruptly and then reached a relative stable value. When the sensors were switched to air again, ammonia desorption happened, and the sensor resistance decreased abruptly and then reached a relative stable value. The response time and recovery time for the VO_2_(B) sensor (defined as 95% of the time between the peak value and the valley value) were 63–85 s and 11–16 s (Figure 7c), respectively. Comparatively, the V_2_O_5_ sensor indicated a faster response: the response time was 14–22 s and the recovery time was 14–20 s (Figure 7d). Compared with other ammonia sensors of VO_2_(B) and V_2_O_5_ reported previously, the sensors in this work showed a fast response/recovery rate (Table 2). As shown in Figure 7c, it is clear that the resistance of the VO_2_(B) sensor fluctuated significantly during the dynamic test and the response time fluctuated in a wide range of 63–85 s, while the V_2_O_5_ sensor showed a slight fluctuation in the resistance and a narrow range in the response time (14–22 s). Clearly, the V_2_O_5_ sensor showed superior reproducibility compared to the VO_2_(B) sensor. In addition, a mass of repeated measurements revealed the response sensitivity of both sensors decreasing after long-term work, and if the sensor was kept in air for about two hours, the response sensitivity would still recover to the original value. This phenomenon can be perfectly interpreted by interactions of surface hydroxyl groups with ammonia. As revealed in previous studies, the humidity in air can improve the ammonia adsorption and resultantly increase the sensor response [46,47]. A long-term work could decrease the site activities of adsorbed hydroxyl groups on the surface, leading to the sensitivity decreasing. After a few hours in air, the ammonia retained on hydroxyl groups should be removed during air purging, thus causing sensitivity recovery.

## 4. Conclusions

In summary, self-assembled VO_2_(B) nanoflakes were synthesized via a simple hydrothermal method, and VO_2_(M) and V_2_O_5_ nanoflakes were obtained through a high-temperature phase transition in nitrogen and air, respectively. Sensors based on the three famous vanadium oxide compounds were fabricated and their gas-sensing characteristics were comparatively investigated at room temperature. It was found that VO_2_(M) nanoflakes were gas-insensitive, while both VO_2_(B) and V_2_O_5_ nanoflakes were highly selective to ammonia. As an ammonia sensor, V_2_O_5_ nanoflakes showed higher sensitivity, faster response, and better reproducibility to ammonia than VO_2_(B) nanoflakes. The sensitivity was about one order of magnitude higher than that of VO_2_(B) nanoflakes, and the response time and recovery time were 14–22 s and 14–20 s, respectively. Interestingly, although vanadium oxides are generally regarded as n-type semiconductors, both VO_2_(B) and V_2_O_5_ nanoflakes showed p-type sensing responses to ammonia, which can be attributed to the surface inversion layer formation.

## Figures and Tables

**Table 1:** Crystal structure, properties, and important applications of VO_2_(B), VO_2_(M), and V_2_O_5_.

Properties of Vanadium Oxides	VO_2_(B)	VO_2_(M)	V_2_O_5_
**Crystal Structure**	Monoclinic (C2/m) *a* = 12.03, *b* = 3.693, *c* = 6.42, *β* = 106.6°	Monoclinic (P21/c) *a* = 5.753, *b* = 4.526, *c* = 5.383, *β* = 122.6°	Orthorhombic (Pmmn) *a* = 11.516, *b* = 3.566, *c* = 4.373
**Structural/Physical/Chemical Characteristics**	Layer structure	Rapid reversible MIT (340 K) Drastic change in resistivity and optical transparency between MIT	Layer structure Oxidizing Enhanced surface reactivity
**Applications**	Energy storage materials Sensors	Chromogenic materials High-speed electronics	Chromogenic materials Catalysts Energy storage materials Sensors

**Table 2:** Comparison of various resistive sensor responses, response and recovery times to ammonia for VO_2_(B) and V_2_O_5_ sensing systems.

Vanadium Oxides	Resistive Response	Response Time	Recovery Time
VO_2_(B) nanoflakes *	0.21 (50 ppm) 5.82 (600 ppm)	63–85 s (550 ppm)	11–16 s (550 ppm)
V_2_O_5_ nanoflakes *	0.24 (50 ppm) 54.4 (600 ppm)	14–22 s (550 ppm)	14–20 s (550 ppm)
VO_2_(B) nanoparticles [38]	0.1 (45 ppm)	310 s	40 min
V_2_O_5_ films [43]	0.419 (40 ppm)	59 s	−
V_2_O_5_ nanobelts [44]	0.7 (100 ppm)	32 s	30 s
V_2_O_5_ fibers [35]	−	50 s (0.85–45 ppm)	350 s (0.85–45 ppm)
V_2_O_5_ nanoparticles [45]	~2.0 (200 ppm)	23 s	13 s

* Indicates vanadium oxide sensor in this work.
